# Supplementary material for: Spatial analysis of malaria hotspots in Dilla sub-watershed: Western Ethiopia
Source: PLoS One. 2023 Apr 6;18(4):e0273271. doi: 10.1371/journal.pone.0273271 (PMC10079056; doi:10.1371/journal.pone.0273271)
Supplement: S1 Text — (DOCX) [file pone.0273271.s001.docx]

**S1 Text: English language Ground Truthing (Observation) data collection checklist**

The observation sites were selected through Simple Random Sampling. Randomness ensures that all parts of the study area would have an equal chance of being sampled without any human bias. Observation sites are selected by dividing the study area into a grid having a number for each row and column. Then any two values are chosen from a random number table, which forms a coordinate pair for defining the location of observation. The coordinate pairs are then plotted on the image which shows the locations where sampling is to be carried out.

**Activities**

It is difficult to give here an exhaustive list of all things to carry while going to ground because they would vary depending upon project objectives. However, an indicative list of requirement is given below:

1) Baseline maps

2) Geo-referenced images

3) Letter of permission/approval to visit the study area

4) Ground truth data sheets and forms

5) GPS and camera with extra batteries

6) Field notebooks, pens, pencils, markers, sharpeners and erasers
