# Supplementary material for: Spatial analysis of malaria hotspots in Dilla sub-watershed: Western Ethiopia
Source: PLoS One. 2023 Apr 6;18(4):e0273271. doi: 10.1371/journal.pone.0273271 (PMC10079056; doi:10.1371/journal.pone.0273271)
Supplement: Attachment — Submitted filename: Review Comments to the Author.docx [file pone.0273271.s002.docx]

The manuscript by Gemechu Y. Ofgeha provides important information of malaria hotspots under the moderate to high malaria transmission setting in Western Ethiopia using meteorological and altitude data as a main source for analysis. I have identified a number of issues that need to be addressed for further strengthening the manuscript as following:

**ABSTRACT (Minor)**

1. I suggest structure abstract which include: **introduction, methods, results and conclusions**
2. *“other primary data”* on line 5 should be listed out.

**INTRODUCTION (Major)**

Generally the introduction part needs major revision. To mention some area of revision

1. Same terms such as “Malaria hotspot”, “Biophysical” etc should be defined. Malaria hotspots occur at micro and macro geographical level, at what level the author wants to conduct malaria hotspot identification?.
2. Many information lack citation
3. The author used outdated data, for example “*Malaria occurs in over 100 countries…World Health Organization estimates that* ***300-500 million cases*** *of malaria occur worldwide* ***each year*** *result the* ***death of over two million people***.” This information also not cited. Another example “… an average of 5 million cases a year and 9.5 million cases per year between 2001 and 2005.” These data are almost 20 years old. The picture of malaria in recent years different in Ethiopia.
4. Most citation of references is not correct. For example, “According to Aynalem, Oromia region is one of the most populous and malaria-prone region in Ethiopia where three-quarters of the administrative districts (242 out of 261) and 3932 kebeles out of 6107 are considered malarious. Seventeen million people are at risk in the region with annual clinical cases numbering between 1.5 and 2 million (Aynalem, 2014).)”. **There is no such information in** “Aynalem, A. (2014). Ethiopian Demography and Health: A Brief Introduction. [www.EthioDemographyAndHealth.Org](http://www.EthioDemographyAndHealth.Org)”
5. The author used the term “incidence” in objective part of abstract and “prevalence” in objective of introduction part. These two words have different meaning the author should be consistent in using such words. He can use both words at the same time in his context.
6. In “**Conceptual framework of the study”**, categorization of variables such as “**Rainfall”, “Slope”, “Proximity to Water Bodies”, and “Soil Moisture Holding Capacity”** need appropriate citation.
7. The author should give equivalent definition of the five agro-ecological climatic zones in English/Amharic (official language of Ethiopia) that he mentioned as “Baddaa *Dilallaa, Baddaa, Badda Daree, Gammojjii and Gammoojjii Ho’aa.”*
8. There are many long, unclear sentences and incorrect grammar. Therefore, the language in the introduction part needs to be improved.
9. The is inappropriate words like “Malaria breeding”

**METHODS AND MATERIALS (Major)**

The methods and material parts are mostly well explained with some relevant uncertainties that have to be addressed. To mentioned some of them

1. “Figure 1: Map of the study Watershed” Need revision. E.g. Name of highlighted region (Oromia Region) in map of Ethiopia should be written in the map. Additionally, in the legend “Ethio_region, Oromia_Zone” should be removed.
2. The author claimed that he collected and used hospital and health center data to substantiate biophysical data, under “**3.2 Research Design”** and **“3.3 Sources and Types of Data”** parts. However, there are no such data in analysis and result parts of the study. If he used such data it needs detail description of the data, e.g. number of hospitals, health centers, and study period, total number of malaria cases and methods of analysis for such data to incorporate in malaria risk map etc.
3. The author also tried to show sources of data and analysis and outcome using a flow chart in “Figure 2”. In this part he mentioned that the key informants, health center data as data source. What data were collected from key informant and health centers? How this data incorporated in the analysis. The figure also misleads the readers.
4. Although the author claimed that the temperature is the same for all study area, I think there should be variation in temperature since as he mentioned altitude ranges between 1317 masl and 2405 masl in the study area.
5. How many years’ average data of temperature and rainfall used for this analysis?
6. At what level (kebele or district) the data available and used for analysis?
7. There was no ethical review and approval in the study. The author stated that he collected data from key informants.

**RESULTS (Major)**

The results are mostly well explained with some relevant uncertainties that have to be addressed. To mentioned some of them

1. In “Table 1” there is only high and very high level category. However, in the result part, the author used additional term “moderately high”. As I mentioned in method part, at what level this data mapped at kebele or districts?
2. The result in the maps in “Figure 2” not logical and convincing. Map in B (Altitude) affects both A (rainfall) and C (temperature). But, areas in South-West has high altitude ranges between 1500 to > 2000masl, but this area has similar rainfall with that of areas

< 1500masl. In C there is no variation in temp across study area. It is difficult to believe the truthfulness of data in this context.

3. I think “Map C” in Figure 3 is not correct. As author mentioned he made a buffer of <1km, 1-2km, and 2-5km. It seems the background map and buffers not proportional.
4. There is error in summation of numbers, e.g sum of areas Km^2^ under variable “Proximity to Rivers” is 1655 not 1650.
5. Under the sub title “**4.2 Malaria Distributions and Risk level of the Watershed”,** there is no data which shows malaria distribution. Therefore, this sub-title needs revision.

**DISCUSSIONS (Major)**

Generally the results poorly discussed, need major revision. To mentioned some points:

1. In the first sentence of discussion author tried to make association of biophysical parameters with spatial variation of malaria prevalence, and compared it with previous studies. However, he didn’t do such analysis and no such results.
2. The author clamed there about “significant difference between the result of spatial hotspot analysis and clinical data of malaria incidences” but he has no such data to discuss this issue.

**CONCLUSION (minor)**

The author recommends distribution of malaria drug, bed net distribution and house spraying for areas identified by his current study. I think without additional information like annual parasite incidence (API) of study area it is difficult to distribute resources only based on malaria risk map.
