# Supplementary material for: Spatial analysis of malaria hotspots in Dilla sub-watershed: Western Ethiopia
Source: PLoS One. 2023 Apr 6;18(4):e0273271. doi: 10.1371/journal.pone.0273271 (PMC10079056; doi:10.1371/journal.pone.0273271)
Supplement: Attachment — Submitted filename: Response to Reviewers.docx [file pone.0273271.s003.docx]

**Response to Reviewers**

**[Cover Letter]**

Dear Editor-in-chief,

I appreciate you and the reviewers for your precious time in reviewing the paper and providing valuable comments. It was your valuable and insightful remarks that led to possible improvements in the current version. I have carefully considered the comments, incorporated the suggestions, and tried my best to address every one of them. I hope the manuscript after careful revisions meet your high standards.

This response letter consists of both general and detailed comments provided by the reviewers. I provide the point-by-point responses for the detailed comments and suggestions. I look forward to hearing from you in due time regarding the resubmission, and to respond to any further questions and comments you may have.

Sincerely,

Gemechu Y. Ofgeha (PhD), corresponding author

[dandigemechu11@gmail.com](mailto:dandigemechu11@gmail.com)

Assistant Professor, Department of Geography and Environmental Studies

Wallagga University, Ethiopia

**Responses**

**Academic Editor**

(Comments #1) Please ensure that your manuscript meets PLOS ONE's style requirements, including those for file naming. The PLOS ONE style templates can be found at

https://journals.plos.org/plosone/s/file?id=wjVg/PLOSOne_formatting_sample_main_body.pdf and https://journals.plos.org/plosone/s/file?id=ba62/PLOSOne_formatting_sample_title_authors_affiliations.pdf

(Response): Thank you very much. All points are very critical that I have incorporated the suggested correction, provide all necessary information in all parts of this paper.

(Comments #2) ……….financial disclosure………

(Response): Thank you very much. All comments are incorporated and the amended statements are explained within cover letter, and included in the sub-section “ethical considerations of the study’

(Comments #3) Please include your full ethics statement in the ‘Methods’ section of your manuscript file. In your statement, please include the full name of the IRB or ethics committee who approved or waived your study, as well as whether or not you obtained informed written or verbal consent. If consent was waived for your study, please include this information in your statement as well.

(Response): Thank you very much. The sub-section “ethical considerations of the study’ is included in the Method section of the manuscript.

(Comments #4) copy right issue for figures

(Response): All data used in the image and figures in this study are open and freely accessible. The biophysical data used in this study were prepared by the Ethiopian Central Statistical Agency. Based on your recommendations, the sources are acknowledged in the caption of the figure.

**Reviewer #1**

(Comment: General) Thank you for the interesting work. A few suggestions are given to improve the readability of the manuscript..

(Response): Thank you very much. I have incorporated the suggested correction in all parts of this paper.

(Comment 1) “The 'kebeles' need to be explained to the international audience, are they a sort of sub-regions/sub-blocks?”

(Response): Thank you very much. Now, the term is defined as footnote.

(Comment 2) “Expansion of the abbreviations like GDP (page 1), CSA(page7), etc should be given at the first mention..”

(Response): Thank you. I accept the suggestion and revised accordingly.

(Comment 3) “Introduction could be more concise and it should substantiate the justification of the study.”

(Response): Thank you for the suggestions. I have made revisions for the introduction and some of the revisions are highlighted.

(Comment 4) “It seems there are many factual errors in the Conceptual Framework section…………………Again while describing elevation, the terms for the local vernacular, seems to rather confuse the readers………….. what is meant by 1500masl?”

(Response): Thank you very much. I accept the suggestion and revised accordingly. 1500masl mean meter above mean sea level. The abbreviation has given at the first mention (Line 206)

(Comment 5) “In figure 1, the component graphs need to be annotated or proper titles to be given.”

(Response): Thank you for the suggestions. The figure caption is amended.

(Comment 5) “Method of data analysis need to be clearly stated, saying the utility function of a proprietary software is not enough.”

(Response): Thank you for the suggestions. The issue is crucial that I have included within cover letter, and explained under sub-section “ethical considerations of the study’ in the Method section of the manuscript.

**Reviewer # 2**

(Comment: ABSTRACT) “I suggest structure abstract which includes: Introduction, Methods, Results and Conclusions “other primary data” on line 5 should be listed out.”

(Response): Thank you for the suggestions. I have made revisions accordingly.

(Comment: INTRODUCTION) “Same terms such as “Malaria hotspot”, “Biophysical” etc should be defined. Malaria hotspots occur at micro and macro geographical level, at what level the author wants to conduct malaria hotspot identification?”

(Response): Thank you very much. The suggested definitions are provided. The level of analysis are clearly indicated.

(Comment: INTRODUCTION) “Many information lack citation.”

(Response): Thank you for the comments. Now, I have made necessary citations in all parts.

(Comment: INTRODUCTION) “The author used outdated data, for example “Malaria occurs in over 100 countries…World Health Organization estimates that 300-500 million cases of malaria occur worldwide each year result the death of over two million people.” This information also not cited. Another example “… an average of 5 million cases a year and 9.5 million cases per year between 2001 and 2005.” These data are almost 20 years old. The picture of malaria in recent years different in Ethiopia)”

(Response): Thank you very for the suggestion. The paragraph and other parts of manuscript with outdated citations are exhaustively reviewed.

(Comment: INTRODUCTION) “The author used the term “incidence” in objective part of abstract and “prevalence” in objective of introduction part. These two words have different meaning the author should be consistent in using such words. He can use both words at the same time in his context.”

(Response): Thank you for the suggestion. Yes, consistence is important and I have revised accordingly.

(Comment: INTRODUCTION) “In Conceptual framework of the study, categorization of variables such as “Rainfall”, “Slope”, “Proximity to Water Bodies”, and “Soil Moisture Holding Capacity” need appropriate citation.”

(Response): Thank you for the suggestion. Now, this part is appropriately cited.

(Comment: INTRODUCTION) “The author should give equivalent definition of the five agro-ecological climatic zones in English/Amharic (official language of Ethiopia) that he mentioned as “Baddaa *Dilallaa, Baddaa, Badda Daree, Gammojjii and Gammoojjii Ho’aa.*”

(Response): Thank you for the suggestion. Now, equivalent definition for each category is given in English. Although Amharic is official language of Ethiopia, using three languages may create redundancy in the manuscript. Therefore, I prefer the two languages by considering local applicability and global audiences.

(Comment: INTRODUCTION) “There are many long, unclear sentences and incorrect grammar. Therefore, the language in the introduction part needs to be improved*.*”

(Response): Thank you very much. Now, I have tried to correct all grammatical errors and lengthy sentence in this revision. However, some corrections are highlighted in the revised manuscript, while highlighting all corrections are a bit difficult.

(Comment: METHODS AND MATERIALS) “Figure 1: Map of the study Watershed” Need revision. E.g. Name of highlighted region (Oromia Region) in map of Ethiopia should be written in the map. Additionally, in the legend “Ethio_region, Oromia_Zone” should be removed*.*”

(Response): Thank you for the suggestion. Now, the map is revised accordingly.

(Comment: METHODS AND MATERIALS) “The author claimed that he collected and used hospital and health center data to substantiate biophysical data, under “3.2 Research Design” and “3.3 Sources and Types of Data” parts. However, there are no such data in analysis and result parts of the study. If he used such data it needs detail description of the data, e.g. number of hospitals, health centers, and study period, total number of malaria cases and methods of analysis for such data to incorporate in malaria risk map etc*.*”

(Response): Thank you for your insightful comments. Before conducting the study, I have tried to observe the cases in the hospital and health centres so as to make sure that the study is relevant/ important. It was a sort of preliminary study, and not used in the analysis. I accept your concern that such explanation should be cancelled. Now, I have cancelled the explanations and the only data used for this study are described in the methods part.

(Comment: METHODS AND MATERIALS) “The author also tried to show sources of data and analysis and outcome using a flow chart in “Figure 2”. In this part he mentioned that the key informants, health center data as data source. What data were collected from key informant and health centers? How this data incorporated in the analysis. The figure also misleads the readers.”

(Response): Thank you once again for the comments. The issue regarding health centre data as data source is similar with the error I have mentioned above. However, during field observation, I have communicated some informants in line with the observations. Therefore, the appropriate way to explain the data source is field observation. The objective of using field observation was not to incorporate the data, rather to check the biophysical data, and provide researcher insights regarding the observable biophysical situation/ground truth.

(Comment: METHODS AND MATERIALS) “Although the author claimed that the temperature is the same for all study area, I think there should be variation in temperature since as he mentioned altitude ranges between 1317 masl and 2405 masl in the study area*.*”

(Response): Thank you for the suggestion. The mean annual temperature was used to construct the temperature data used in this study. If mean monthly, seasonal or lower time scale are used, the spatial difference in temperature could be observed. However, by this data, the same temperature is observed.

(Comment: METHODS AND MATERIALS) “How many years’ average data of temperature and rainfall used for this analysis?”

(Response): The most recent available temperature and rainfall data was used. The objective is to examine the current situation of malaria hot spots in the study area. In think, the longitudinal data may be applicable to understand the dynamics over time and associated impacts on malaria transmissions.

(Comment: METHODS AND MATERIALS) “At what level (kebele or district) the data available and used for analysis?”

(Response): All data used in this study are raster data which could be masked at any geographical and administrative level. For better application by any interested users, the kebele and district based administrative shape file of the study watershed were used.

(Comment: METHODS AND MATERIALS) “There was no ethical review and approval in the study. The author stated that he collected data from key informants.”

(Response): Thank you for the suggestion. Now, the ethical issue is considered and stated as separate sub-section under methods and materials.

(Comment: Results) “In “Table 1” there is only high and very high level category. However, in the result part, the author used additional term “moderately high”. As I mentioned in method part, at what level this data mapped at kebele or districts? ”

(Response): The value in the table 1 is the separate value of each and every variables which ranges from 1 to 5 (stands from very low risk to very high risks respectively). Since it is standardized value for malaria risks, the watershed may have all value (risk level) for one variable, three for the other, two for the other and etc depending on the standardized values assigned for all risk level (Please, check in the table 3). However, the result part (Particularly, figure 4) is based on the weighted overlie analysis by which all variables are overlaid to produce malaria risk map. Similar to previous research procedures, I am interested to identify the areas of high (very high and high masked together) and moderate risk areas by avoiding very low and low risk area.

(Comment: Results) “The result in the maps in “Figure 2” not logical and convincing. Map in B (Altitude) affects both A (rainfall) and C (temperature). But, areas in South-West has high altitude ranges between 1500 to > 2000masl, but this area has similar rainfall with that of areas

< 1500masl. In C there is no variation in temp across study area. It is difficult to believe the truthfulness of data in this context.”

(Response): Thank you for the suggestion. The standardized value for malaria risk level indicated in the table 1 is the criteria in the classification. For instance, the areas within 1500-2000m may show difference rainfall as you mentioned in this comment. However, are labelled as the same in the malaria risk prevalence as far rain is concerned. This standard was adopted from previous malaria studies. The study area is a sub-watershed (couldn’t represent the situation in south-western Ethiopia as whole).

(Comment: Results) “There is error in summation of numbers, e.g sum of areas Km2 under variable “Proximity to Rivers” is 1655 not 1650.”

(Response): Thank you for the in-depth review of the manuscript. I have corrected in this revision.

(Comment: Results) “Under the sub title “4.2 Malaria Distributions and Risk level of the Watershed”, there is no data which shows malaria distribution. Therefore, this sub-title needs revision.”

(Response): Thank you for the suggestion. The first sub-section of the result (4.1) describes the malaria risk level by associating with the variable separately. However, in the 4.2 the weighted overlie analysis was made to show the aggregate impacts of the variables Malaria (Distributions and Risk level of the Watershed).

(Comment: Discussions) “In the first sentence of discussion author tried to make association of biophysical parameters with spatial variation of malaria prevalence, and compared it with previous studies. However, he didn’t do such analysis and no such results.”

(Response): Thank you for the comments. The objective of the study is to identify the areas in the watershed potentially affected by malaria due to their biophysical characteristics. Actually, other data such as clinical data could be used to substantiate the results, while in this study the data was not used (except field observation). However, the previous studies those used both data come up with the strong association.

(Comment: Discussions) “The author clamed there about “significant difference between the result of spatial hotspot analysis and clinical data of malaria incidences” but he has no such data to discuss this issue.”

(Response): Thank you for the comment. The sentence was wrong and now I have corrected as follow: The previous studies those used both historical clinical data of malaria incidences and geospatial models confirm a similar pattern and strong positive association between the clinical admissions and biophysical based hot spot situations (29,30).

(Comment: Conclusions) The author recommends distribution of malaria drug, bed net distribution and house spraying for areas identified by his current study. I think without additional information like annual parasite incidence (API) of study area it is difficult to distribute resources only based on malaria risk map.

(Response): Thank you for the comment. Yes, this study is not sufficient to understand the vulnerability situations, and I have indicated that comprehensive vulnerability study is important to capture the local sensitivity and adaptation capacity which are the function of socioeconomic characteristics of the community. This study may be applicable in the absence of such comprehensive studies, and provide an input for future research.
