# Supplementary material for: Spatial analysis of malaria hotspots in Dilla sub-watershed: Western Ethiopia
Source: PLoS One. 2023 Apr 6;18(4):e0273271. doi: 10.1371/journal.pone.0273271 (PMC10079056; doi:10.1371/journal.pone.0273271)
Supplement: Attachment — Submitted filename: Response to Reviewers.docx [file pone.0273271.s004.docx]

**Response to Reviewers**

**[Cover Letter]**

Dear Editor-in-chief,

I appreciate you and the reviewers for your precious time in reviewing the paper and providing valuable comments. It was your valuable and insightful remarks that led to possible improvements in the current version. I have carefully considered the comments, incorporated the suggestions, and tried my best to address every one of them. I hope the manuscript after careful revisions meet your high standards.

This response letter consist the responses on the comments provided by the editor and reviewers. I provide the point-by-point responses for the detailed comments and suggestions. I look forward to hearing from you in due time regarding the resubmission, and to respond to any further questions and comments you may have.

Sincerely,

Gemechu Y. Ofgeha (PhD), corresponding author

[dandigemechu11@gmail.com](mailto:dandigemechu11@gmail.com)

Assistant Professor, Department of Geography and Environmental Studies

Wallagga University, Ethiopia

**Responses**

**Academic Editor**

(Comments) Journal Requirements: Please review your reference list to ensure that it is complete and correct. If you have cited papers that have been retracted, please include the rationale for doing so in the manuscript text, or remove these references and replace them with relevant current references. Any changes to the reference list should be mentioned in the rebuttal letter that accompanies your revised manuscript. If you need to cite a retracted article, indicate the article’s retracted status in the References list and also include a citation and full reference for the retraction notice. (Response): Thank you very much. All points are very critical that I have incorporated the suggested correction, provide all necessary information in all parts of this paper.

(Response): Thank you very much. I have exhaustively reviewed the reference list that it is complete and correct; and no retracted citation was used in this manuscript. Because of some revisions mainly to trim the introduction part, new citations are used, and referenced.

- Devi P, B M, S B. Use of Remote Sensing and GIS for Monitoring the Environmental Factors Associated With Vector-Borne Disease (Malaria). Proc Third Int Conference Environment
- Gebreslasie MT. A review of spatial technologies with applications for malaria transmission modelling and control in Africa. Geospat Health. 2015;10(2):239–47.
- Larocca A, Moro Visconti R, Marconi M. Malaria diagnosis and mapping with m-Health and geographic information systems (GIS): evidence from Uganda. Malar J. 2016; 15(1):1–12.

**Reviewer #1**

(Comment) “The manuscript has substantially improved. Still the language does not have professional vibe. It would be better to get your manuscript, vetted by a language editor. The introduction session could be trimmed”

(Response): Thank you for your positive response on the revision. I have carefully considered the suggestions to improve the language problems. Now, I have used the English language profession in the host University for this ‘Revision’. True to the critical comments raised by the respected reviewer, I have found several grammatical errors. However, now the manuscript is carefully revised. The introduction part is exhaustively revised and trimmed.
